# Gender differences in the association between dietary protein intake and constipation: findings from NHANES

**DOI:** 10.3389/fnut.2024.1393596

**Published:** 2024-06-19

**Authors:** Yongping Hong, Hongchen Shen, Xingxing Chen, Guofeng Li

**Affiliations:** ^1^Department of Anorectal Surgery, The First People’s Hospital of Xiaoshan District, Xiaoshan Affiliated Hospital of Wenzhou Medical University, Hangzhou, China; ^2^The Second Department of Medicine, Renji College of Wenzhou Medical University, Wenzhou, China; ^3^Department of Clinical Research, The First People’s Hospital of Xiaoshan District, Xiaoshan Affiliated Hospital of Wenzhou Medical University, Hangzhou, China

**Keywords:** protein intake, constipation, stool consistency, cross-sectional study, NHANES

## Abstract

**Purpose:**

Dietary factors play a crucial role in the development and management of chronic constipation, yet the relationship between dietary protein intake and constipation remains underexplored. This study aims to investigate the association between dietary protein intake and the prevalence of constipation among American adults, with a focus on potential gender differences, using large-scale national data.

**Materials and methods:**

Data from 14,048 participants aged 20 and above (7,072 men and 6,976 women) from the National Health and Nutrition Examination Survey (NHANES) 2005–2010 were analyzed. The Bristol Stool Form Scale’s types 1 (separate hard lumps, resembling nuts) and 2 (sausage-shaped, but lumpy) were used to define constipation. A 24-h dietary recall technique was used to measure dietary protein intake. After controlling for covariates, the association between protein consumption and constipation risk was examined using multivariable logistic regression, smooth curve fitting, and testing for gender interaction effects. We then further determined the threshold effect between dietary protein intake and constipation risk.

**Results:**

Constipation was present in 7.49% of people overall, with a higher proportion among women (10.19%) than among males (4.82%). In men, higher protein intake was significantly associated with a lower rate of constipation. However, in women, higher protein intake correlated with an increased risk of constipation, and the interaction between gender was significant (*P* for interaction = 0.0298). These results were corroborated by smooth curve fits, which also demonstrated a dose–response effect. Further threshold effect analysis showed that the turning points of dietary protein intake differed between male and female participants (119.42 gm/day for men; 40.79 gm/day for women).

**Conclusion:**

The association between dietary protein intake and constipation was different in different genders with threshold effect. For men, moderately increasing protein intake could be beneficial, while for women, exceeding a certain level may increase the risk of constipation. These insights are crucial for guiding dietary protein recommendations for different genders and have significant clinical implications.

## Introduction

Chronic constipation is a frequent gastrointestinal condition that significantly impacts patients’ quality of life. It affects an estimated 2 to 27% of adults in western countries (1), with a global prevalence of approximately 14% (2). Women are more affected than men (3), especially among the elderly (4). The etiology of chronic constipation is complex and not fully understood, with factors such as diet, lifestyle, gut microbiota, and underlying diseases playing roles in its development (5). However, differences in treatment approaches result in suboptimal efficacy, underscoring the importance of effective management for improving patient outcomes.

With special focus to the connection between dietary components and chronic constipation, dietary determinants are thought of as modifiable risk factors for this illness. Soluble fiber and trace minerals including phosphorus, magnesium, and selenium have been demonstrated in earlier studies to lower the risk of chronic constipation (6–8), but women who consume high levels of saturated fat or relatively modest amounts of energy are at higher risk (9, 10). Thus, research into possible dietary approaches for treating chronic constipation provides promise for the illness’s management and prevention.

Protein is ubiquitous in the human diet, and adequate protein intake is crucial for maintaining health. Research indicates that dietary protein levels can affect intestinal enzyme activity, microbiota composition, and immune response (11–13). Additionally, diseases such as protein-losing enteropathy highlight the impact of protein on specific intestinal disorders (14). However, research on the influence of dietary protein intake on chronic constipation is limited. Thus, this study aims to determine the incidence of chronic constipation among United States (US) adults and investigate the relationship between dietary protein intake and constipation, focusing on both women and men, using data from the National Health and Nutrition Examination Survey (NHANES) database.

## Materials and methods

### Study population

The National Center for Health Statistics of the Centers for Disease Control and Prevention conducts the National Health and Nutrition Examination Survey, a cross-sectional survey that assesses the health and nutrition of the US population. The survey employs a stratified multi-stage sampling design. All participants gave written consent. Our study utilized publicly available data from the 2005–2006, 2007–2008, and 2009–2010 NHANES cycles, as gut health information was only available for these periods.

**Figure 1:**
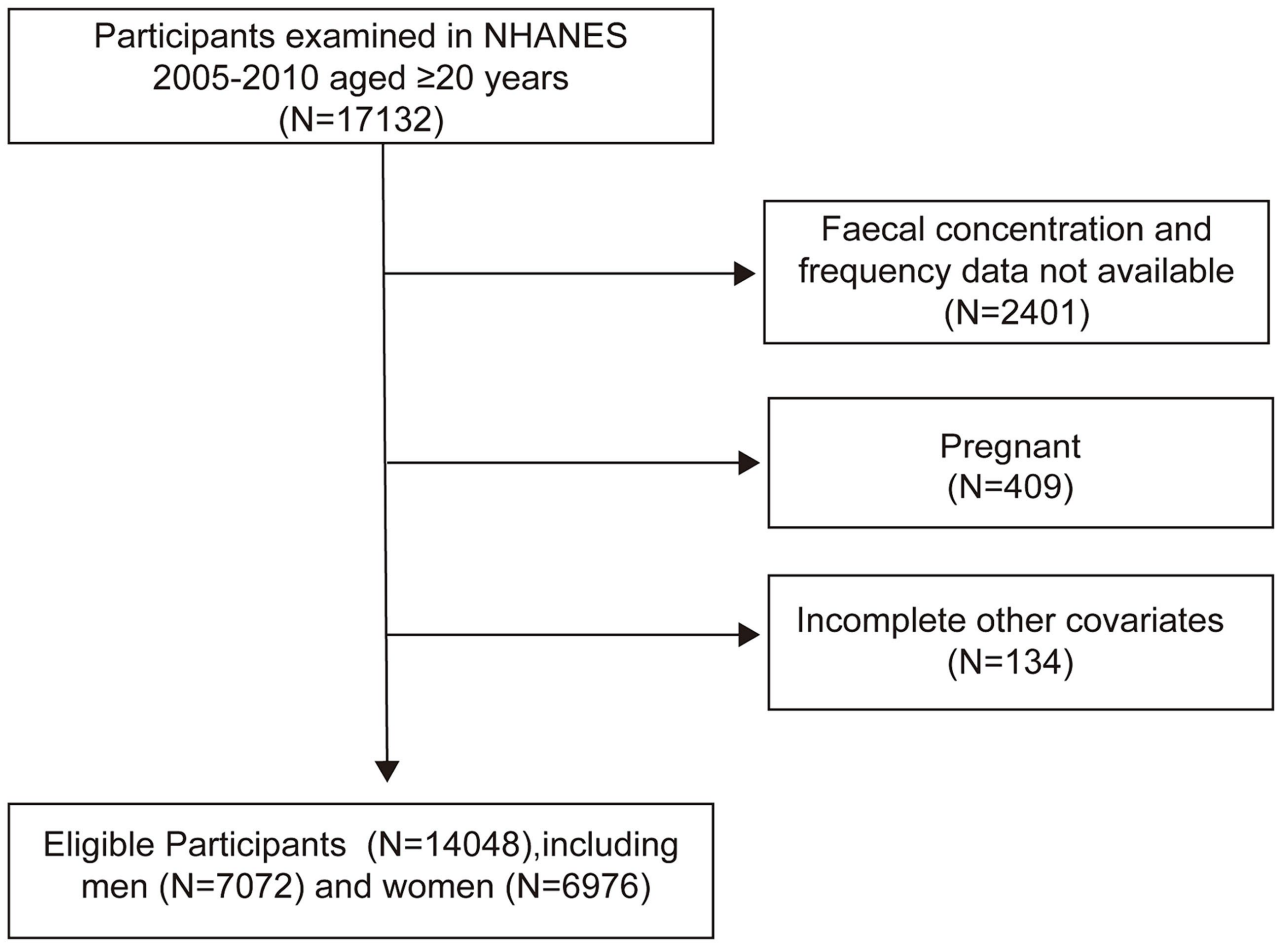
Flowchart of the selection process.

From the 2005–2010 NHANES dataset, we included 17,132 participants aged 20 years and older. Excluding 2,401 participants with incomplete stool data, 407 pregnant women, and 134 with insufficient confounder information, our final cohort comprised 14,048 individuals (7,072 men and 6,976 women) (Figure 1).

### Definition of constipation

Constipation was defined by stool frequency and consistency, which were noted in the NHANES gut health questionnaire from 2005 to 2010. We preferred the Bristol Stool Form Scale (BSFS), which is used to assess stool consistency during Mobile Examination Center (MEC) interviews for persons aged 20 and over, based on previous studies that estimated constipation in the NHANES surveys. Using a graphic card, participants identified the sort of stool they typically use. Constipation was defined as BSFS type 1 (separate hard lumps, like nuts) or type 2 (sausage-like, but lumpy). Normal stool consistency was classified as BSFS type 3 (like a sausage but with cracks in the surface), type 4 (like a sausage or snake, smooth and soft), or type 5 (soft blobs with clear-cut edges). BSFS type 6 (fluffy chunks with ragged edges, a mushy stool) or type 7 (watery, no solid pieces) was used to define chronic diarrhea.

### Dietary measures

Dietary protein intake for men and women was determined through two 24-h dietary recalls. The first interview was conducted at the MEC, followed by a telephone interview 3–10 days later. The survey provided detailed information on the nutrient content of each food item consumed. Dietary protein intake for each participant was averaged based on 2 days of dietary recall data when available, or otherwise based on a single day’s data.

Additionally, we collected data on other dietary variables potentially related to constipation, such as energy, carbohydrates, total sugars, dietary fiber, total fat, cholesterol, and moisture intake, and calculated their averages as confounding factors.

### Other covariates

We also considered potential confounding factors such as age, race/ethnicity, education level, marital status, income-poverty ratio, body mass index (BMI), physical activity, smoking, alcohol consumption, diabetes, hypertension, and milk consumption in our analysis.

Age was categorized into three groups: under 45, 45–65, and over 65 years. Race/ethnicity was classified as Non-Hispanic White, Non-Hispanic Black, Mexican American, and Others. Education level was divided into less than high school and high school or higher, marital status into never married, widowed/divorced/separated, and married, and family income-to-poverty ratio into two groups: less than 2 and 2 or greater. Underweight or normal (BMI < 25), overweight (BMI 25–30), and obese (BMI > 30) were the three BMI categories.

We calculated the metabolic equivalent of task (MET) hours per week by multiplying the time spent on each activity per week by its MET value. For cycles after 2007, due to changes in the questionnaire, we estimated weekly MET minutes by multiplying the minutes spent in moderate and vigorous activities by their respective MET values. According to the U.S. Department of Health and Human Services, participants with a weekly MET value of ≥500 were classified as physically active, while those with <500 were considered inactive. Smoking status was categorized into never smokers (those who never smoked or smoked less than 100 cigarettes in their lifetime), current smokers (those who have smoked ≥100 cigarettes in their lifetime and are currently smoking), and former smokers (those who smoked <100 cigarettes but no longer smoke). Drinkers were defined as individuals consuming at least 12 drinks per year.

Diabetes was defined as having been diagnosed by a doctor or having a glycated hemoglobin level of 6.5% or higher. Hypertension was classified as having been diagnosed by a doctor, taking medication for high blood pressure, or having systolic blood pressure greater than 130 mmHg or diastolic blood pressure greater than 80 mmHg. Milk consumption was divided into four categories: never, rarely (less than once a week), occasionally (once a week or more), and frequently (once a day or more).

### Statistical analysis

Considering NHANES’ complex sampling design, we appropriately weighted the data (one-third of the 2005–2010 weights) according to NHANES guidelines. Categorical variables were analyzed using weighted χ2 tests, and continuous variables were analyzed using weighted linear regression models.

We investigated the association between dietary protein intake and chronic constipation in both genders using multivariate logistic regression analysis. Participants were categorized into quintiles based on their protein intake distribution for both genders, with an analysis of trends across these groups. Model 1 was unadjusted, whereas Model 2 was adjusted for confounders such as age, race/ethnicity, education, marital status, and income-poverty ratio, all treated as continuous variables. Model 3 additionally adjusted for BMI, physical activity, smoking status, alcohol consumption, diabetes, hypertension, and dietary factors including energy, carbohydrates, sugars, fiber, fat, cholesterol, and moisture intake. Interaction tests were employed to explore the heterogeneity in the effects of dietary protein intake on chronic constipation between genders.

After completely controlling for variables, we used generalized additive models (GAM) to build weighted smoothing curves in order to better examine the association between dietary protein intake and the risk of constipation. After identifying nonlinearities, we used a recursive approach to determine the threshold effect between dietary protein intake and constipation risk. R (http://www.Rproject.org) and EmpowerStats (http://www.empowerstats.com) were used for all analyses, and *p* < 0.05 was chosen as the significant level.

## Results

The study involved 14,048 adults, comprising 7,072 men and 6,976 women. Table 1 presents the baseline characteristics of the participants. Overall, constipation was more prevalent in women (10.19%, 711/6976) than in men (4.82%, 341/7072). Regardless of gender, constipation was more prevalent among Non-Hispanic White individuals (both *p* < 0.01), those with lower educational levels (both *p* < 0.001), and those with lower income-poverty ratio (both *p* < 0.05). Constipated individuals, both men and women, had significantly lower total energy intake, dietary fiber, total fat, moisture, and protein intake (all *p* < 0.05). Women with constipation had higher total sugar intake (*p* = 0.026). Additionally, men with constipation had lower levels of physical activity and a higher proportion in the lowest quartile of protein intake (both *p* < 0.001). Interestingly, while constipation has traditionally been associated with age, the prevalence of constipation in women was significantly higher under 45 years of age (*p* = 0.018), with no significant differences observed among age groups in constipated men (*p* = 0.209).

**Table 1:** Participant characteristics by constipation among men and women from National Health and Nutrition Examination Surveys (NHANES) 2005–2010.

Characteristics	Male (*N* = 7,072)		*p* value	Female (*N* = 6,976)		*p* value
	No constipation (*N* = 6,731)	Constipation (*N* = 341)		No constipation (*N* = 6,265)	Constipation (*N* = 711)	
Age (years)			0.209			0.018
< 45	2,807 (41.70%)	158 (46.33%)		2,574 (41.09%)	330 (46.41%)	
≥ 45, < 65	2,267 (33.68%)	102 (29.91%)		2,188 (34.92%)	218 (30.66%)	
≥ 65	1,657 (24.62%)	81 (23.75%)		1,503 (23.99%)	163 (22.93%)	
Race/ethnicity			<0.001			0.008
Non-Hispanic white	3,406 (50.60%)	134 (39.30%)		3,075 (49.08%)	322 (45.29%)	
Non-Hispanic black	1,325 (19.69%)	78 (22.87%)		1,230 (19.63%)	168 (23.63%)	
Mexican American	1,214 (18.04%)	84 (24.63%)		1,136 (18.13%)	111 (15.61%)	
Others	786 (11.68%)	45 (13.20%)		824 (13.15%)	110 (15.47%)	
Education			<0.001			<0.001
< High school	3,559 (52.91%)	238 (69.79%)		3,110 (49.69%)	405 (57.20%)	
≥ High school	3,167 (47.09%)	103 (30.21%)		3,149 (50.31%)	303 (42.80%)	
Marital status			0.115			0.115
Never married	1,695 (25.20%)	96 (28.15%)		1,415 (22.60%)	183 (25.74%)	
Widowed/divorced/separated	3,950 (58.72%)	181 (53.08%)		3,015 (48.16%)	318 (44.73%)	
Married	1,082 (16.08%)	64 (18.77%)		1831 (29.24%)	210 (29.54%)	
Income-poverty ratio			<0.001			0.015
< 2	2,681 (42.92%)	180 (56.25%)		2,758 (47.53%)	342 (52.53%)	
≥ 2	3,566 (57.08%)	140 (43.75%)		3,045 (52.47%)	309 (47.47%)	
BMI (kg/m^2^)			0.070			<0.001
< 25	1779 (26.43%)	108 (31.67%)		1877 (29.96%)	262 (36.85%)	
25–30	2,657 (39.47%)	132 (38.71%)		1833 (29.26%)	215 (30.24%)	
> 30	2,295 (34.10%)	101 (29.62%)		2,555 (40.78%)	234 (32.91%)	
Physical activity			0.021			0.907
< 500	2080 (34.62%)	123 (41.14%)		2,426 (43.16%)	273 (43.40%)	
≥ 500	3,928 (65.38%)	176 (58.86%)		3,195 (56.84%)	356 (56.60%)	
Drinking			<0.001			<0.001
No	1,086 (16.15%)	78 (22.94%)		2,440 (38.97%)	328 (46.20%)	
Yes	5,640 (83.85%)	262 (77.06%)		3,822 (61.03%)	382 (53.80%)	
Smoking			0.069			0.351
Never	3,085 (45.85%)	178 (52.20%)		3,616 (57.74%)	428 (60.20%)	
Current	1,676 (24.91%)	73 (21.41%)		1,268 (20.25%)	142 (19.97%)	
Former	1967 (29.24%)	90 (26.39%)		1,379 (22.02%)	141 (19.83%)	
Diabetes			0.041			0.734
No	5,713 (85.10%)	303 (89.12%)		5,315 (85.01%)	607 (85.49%)	
Yes	1,000 (14.90%)	37 (10.88%)		937 (14.99%)	103 (14.51%)	
Hypertension			0.183			0.026
No	3,213 (48.21%)	176 (51.92%)		3,208 (51.80%)	392 (56.24%)	
Yes	3,451 (51.79%)	163 (48.08%)		2,985 (48.20%)	305 (43.76%)	
Energy (kcal/d)	2385.51 ± 950.89	2220.17 ± 968.36	0.002	1724.46 ± 628.10	1686.55 ± 603.96	0.128
Carbohydrate (gm/d)	285.56 ± 122.02	276.45 ± 118.48	0.182	216.94 ± 85.70	217.87 ± 84.10	0.783
Total sugars (gm/d)	126.77 ± 77.42	128.77 ± 81.02	0.646	99.85 ± 55.58	104.77 ± 55.15	0.026
Dietary fiber (gm/d)	17.84 ± 9.52	15.94 ± 8.56	<0.001	14.69 ± 7.35	13.57 ± 6.97	<0.001
Total fat (gm/d)	88.91 ± 44.11	80.69 ± 47.62	<0.001	64.52 ± 30.11	61.84 ± 28.87	0.025
Cholesterol (mg/d)	341.04 ± 221.39	311.03 ± 229.46	0.016	232.71 ± 148.46	231.03 ± 156.01	0.777
Moisture (gm/d)	3048.26 ± 1372.54	2685.71 ± 1239.50	<0.001	2521.52 ± 1083.51	2334.12 ± 1036.01	<0.001
Protein (gm/d)	93.95 ± 40.04	83.57 ± 39.40	<0.001	66.99 ± 26.04	64.95 ± 26.10	0.048
Quintile Protein intake			<0.001			0.282
Lower quintile	711 (10.74%)	67 (20.00%)		1761 (28.57%)	225 (31.96%)	
Lower-middle quintile	929 (14.04%)	57 (17.01%)		1,591 (25.82%)	187 (26.56%)	
Middle quintile	1,234 (18.65%)	62 (18.51%)		1,328 (21.55%)	139 (19.74%)	
Upper-middle quintile	1,586 (23.96%)	71 (21.19%)		1,005 (16.31%)	103 (14.63%)	
Upper quintile	2,158 (32.61%)	78 (23.28%)		478 (7.76%)	50 (7.10%)	

Mean ± SD for continuous variables: the *P* value was calculated by the weighted linear regression model. (%) for categorical variables: the p value was calculated by the weighted chi-square test.

Table 2 presents the results of the multiple regression analysis. In the unadjusted model 1 for men, a higher quartile of protein intake was significantly inversely associated with the constipation risk, showing a decreasing trend in odds ratios (ORs) from the first to the fifth quartile (all *p* < 0.05). In Q5 men, the ORs of constipation was only 0.38 times that of Q1 (95% CI: 0.27, 0.54, *p* < 0.0001), with a significant overall trend (*p* < 0.001). Even after adjusting for sociodemographic and lifestyle factors in model 2 (*P* for trend <0.001) and model 3 (*P* for trend = 0.047), a negative correlation between high dietary protein intake and low constipation risk persisted. In the fully adjusted model 3, the ORs of constipation in Q5 remained 0.50 times that of Q1 (95% CI: 0.28, 0.91, *p* = 0.0235).

**Table 2:** The association between the quintiles of dietary protein intake and constipation among men and women.

	Model 1 OR (95% CI) *p* value	Model 2 OR (95% CI) *p* value	Model 3 OR (95% CI) *p* value
**Men**			
Q1 (8.71–51.41 gm/d)	Reference	Reference	Reference
Q2 (51.42–66.26 gm/d)	0.65 (0.45, 0.94) 0.0217	0.63 (0.43, 0.93) 0.0206	0.69 (0.45, 1.07) 0.0998
Q3 (66.27–82.31 gm/d)	0.53 (0.37, 0.76) 0.0006	0.58 (0.40, 0.84) 0.0042	0.68 (0.44, 1.05) 0.0838
Q4 (82.32–104.13 gm/d)	0.48 (0.34, 0.67) <0.0001	0.49 (0.34, 0.71) 0.0001	0.64 (0.40, 1.02) 0.0578
Q5 (104.14–474.01 gm/d)	0.38 (0.27, 0.54) <0.0001	0.39 (0.27, 0.56) <0.0001	0.50 (0.28, 0.91) 0.0235
*P* for trend	<0.001	<0.001	0.047
**Women**			
Q1 (3.46–51.42 gm/d)	Reference	Reference	Reference
Q2 (51.43–66.28 gm/d)	0.92 (0.75, 1.13) 0.4258	0.98 (0.79, 1.22) 0.8756	1.24 (0.95, 1.62) 0.1174
Q3 (66.29–82.30 gm/d)	0.82 (0.66, 1.02) 0.0798	0.87 (0.68, 1.10) 0.2374	1.22 (0.87, 1.72) 0.2405
Q4 (82.31–104.08 gm/d)	0.80 (0.63, 1.03) 0.0786	0.88 (0.68, 1.14) 0.3391	1.54 (1.02, 2.34) 0.0423
Q5 (104.09–299.83 gm/d)	0.82 (0.59, 1.13) 0.2243	0.89 (0.63, 1.24) 0.4742	2.00 (1.09, 3.68) 0.0247
*P* for trend	0.055	0.246	0.024
*P* interaction	0.0236	0.0102	0.0279

Model 1: no covariates were adjusted.

Model 2: age, race/ethnicity, education, marital status, and income-poverty ratio were adjusted.

Model 3: age, race/ethnicity, education, marital status, income-poverty ratio, body mass index, physical activity, drinking and smoking behavior, the existence of diabetes, hypertension, and dietary intake of energy, carbohydrate, total sugars, dietary fiber, total fat, cholesterol, and moisture were adjusted.

However, in models 1 and 2 for women, there was no significant distinction in constipation risk among quartiles (all *p* > 0.05). After comprehensive adjustment for covariates in model 3, women in Q5 had a significantly higher risk of constipation compared to Q1 (OR = 2.00, 95% CI: 1.09, 3.68, *p* = 0.0247), with a significant effect observed also in Q4 (OR = 1.54, 95% CI: 1.02, 2.34, *p* = 0.0423). There was a significant increasing trend in ORs for constipation among quartiles in model 3 (*P* for trend = 0.024), with a significant interaction between genders (*P* for interaction = 0.0279).

**Figure 2:**
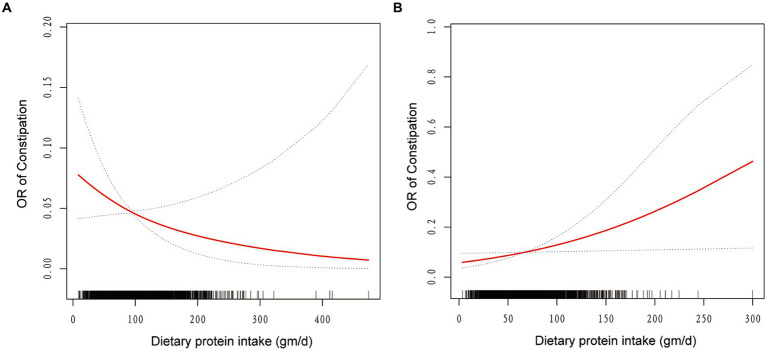
The association between dietary protein intake and odds ratio of constipation among men **(A)** and women **(B)**. Solid red line represents the smooth curve fit between variables. Blue bands represent the 95% of confidence interval from the fit. Age, race/ethnicity, education, marital status, income-poverty ratio, body mass index, physical activity, drinking and smoking behavior, the existence of diabetes, hypertension, and dietary intake of energy, carbohydrate, total sugars, dietary fiber, total fat, cholesterol, and moisture were adjusted.

Weighted smooth curve fittings based on GAM have further demonstrated the dose–response relationship between dietary protein intake and the risk of chronic constipation for both genders. As illustrated in Figure 2, there is a negative dose–response relationship between protein intake and the OR of constipation in men (Figure 2A), whereas a positive dose–response relationship is observed in women (Figure 2B). As shown in Table 3, further analysis of threshold effects revealed that in male study participants, the turning point for dietary protein intake is 119.42 gm/day. Below this threshold, each additional 1 mg/day of protein intake decreases the risk of constipation by 1% (OR = 0.99, 95% CI: 0.98–1.00, *p* = 0.0493), showing a significant downward trend. However, above this threshold of 119.42 gm/day, there is no significant association between dietary protein intake and constipation risk (OR = 1.00, 95% CI: 0.99–1.01, *p* = 0.8273). For women, the turning point for dietary protein intake is 40.79 gm/day. Below this threshold, there is no significant correlation between dietary protein intake and constipation risk (OR = 1.00, 95% CI: 0.97–1.02, *p* = 0.7230). However, exceeding 40.79 gm/day, each additional 1 mg/day of protein intake increases the risk of constipation by 1%, reaching statistical significance (OR = 1.01, 95% CI: 1.00–1.02, *p* = 0.0199).

**Table 3:** Threshold effect analysis of dietary protein intake on the risk of constipation in men and women.

Constipation	OR (95% CI), *P* value
**Men**	
Inflection point	119.42
Dietary protein intake <119.42 (gm/day)	0.99 (0.98, 1.00) 0.0493
Dietary protein intake >119.42 (gm/day)	1.00 (0.99, 1.01) 0.8273
**Women**	
Inflection point	40.79
Dietary protein intake <40.79 (gm/day)	1.00 (0.97, 1.02) 0.7230
Dietary protein intake >40.79 (gm/day)	1.01 (1.00, 1.02) 0.0199

Age, race/ethnicity, education, marital status, income-poverty ratio, body mass index, physical activity, drinking and smoking behavior, the existence of diabetes, hypertension, and dietary intake of energy, carbohydrate, total sugars, dietary fiber, total fat, cholesterol, and moisture were adjusted.

## Discussion

For the first time, we evaluated the association between dietary protein intake and chronic constipation in an adult population that was nationally representative. Constipation symptoms include hard stools, irregular bowel motions, straining, and pain in the abdomen (15). Previous studies have indicated that stool consistency, as described by the validated BSFS, is recommended over bowel frequency as a more effective measure for defining constipation due to its better correlation with colon transit time (16). According to stool consistency, the overall prevalence of constipation in our study was 7.49%; women were more likely to experience it (10.19%) than men (4.82%). Despite being slightly lower than the global prevalence reported due to differences in populations and definitions, the distribution of risk between genders was consistent (3).

This research unveils for the first instance the distinct associations of dietary protein consumption with constipation in adult males and females. In adult men, an increased dietary protein intake correlates with a reduced risk of chronic constipation. This negative dose–response relationship remained significant even after adjusting for potential confounding factors, including age, ethnicity, education level, marital status, income-to-poverty ratio, alcohol consumption, smoking, diabetes status, hypertension, BMI, physical activity, and dietary intake. However, in females, the initial analysis did not show a clear connection between protein intake and constipation due to confounding factors. Surprisingly, after fully adjusting for these factors, we found that higher dietary protein intake was associated with a higher risk of chronic constipation, demonstrating a significant positive dose–response relationship. The difference in the association between dietary protein and chronic constipation risk between genders was statistically significant. This crucial discovery enhances our understanding of how dietary factors affect intestinal health differently in men and women, which is important for making personalized dietary recommendations.

A small cross-sectional descriptive study on children over 4 years old showed no significant difference in average daily protein intake between the constipation and control groups (17). This inconsistency with the results of our study may be caused by the small sample size and the fact that the study population mainly targeted minors. One study of the role of a low-protein diet in managing chronic kidney disease patients mentioned that a plant-based low-protein diet might reduce the risk of constipation, but did not elaborate on the mechanism (18). Another study indicated that increasing protein intake could indirectly affect constipation symptoms and quality of life by improving the nutritional status and physical function of chronic obstructive pulmonary disease (COPD) patients (19). These prior studies suggest that the mechanism of dietary protein’s role in intestinal health may vary across different populations.

Our study uniquely provides insights into the gender-specific effects of dietary protein on chronic constipation, a topic not extensively explored in other large-scale epidemiological studies. Several mechanisms may explain our findings. Firstly, dietary protein could differently affect the gut microbiome composition in males and females, favoring different bacterial phyla, which in turn may influence constipation risk oppositely across genders (20, 21). Secondly, the breakdown of amino acids in proteins leads to the production of metabolic byproducts such as ammonia, branched-chain fatty acids (BCFAs), and short-chain fatty acids (SCFAs). These byproducts can alter the pH of feces and change its consistency (22, 23). Hormonal differences between genders can further regulate these effects. Hormonal fluctuations may influence protein metabolism through gut microbiota, resulting in gender-specific impacts on fecal characteristics (24). Additionally, proteins supply amino acids for muscle, and since men typically have greater muscle mass, this could enhance intestinal motility and transport, reducing constipation risk (25). In the United States, the average daily protein intake is approximately 82 grams for men and 67 grams for women (26). This indicates that men generally consume more protein than women, reflecting gender differences in protein requirements. Excessive protein intake, particularly for women, can lead to an imbalance in intestinal and microbial metabolism, thereby increasing the risk of constipation (27, 28).

This study delves deeper into the threshold effect between dietary protein intake and the risk of constipation, which varies between males and females. For men, the threshold is set at 119.42 gm/day. Below this level, an increase in dietary protein significantly reduces the risk of constipation; above this level, no significant correlation is observed. For women, the threshold is 40.79 gm/day. Below this threshold, protein intake does not significantly correlate with constipation; however, exceeding this threshold leads to a significant increase in constipation risk. These findings suggest that optimal dietary protein intake to reduce constipation risk may differ by gender. For men, moderately increasing protein intake could be beneficial, while for women, exceeding a certain level may increase the risk of constipation. These insights are crucial for guiding dietary protein recommendations for different genders and have significant clinical implications.

There are various advantages to this research. To our knowledge, it fills a vacuum in the literature by evaluating for the first time, using the most nationally representative sample, the association between adult dietary protein intake and constipation. It’s also the first study to show how protein intake and constipation differ in men and women, offering a useful benchmark for dietary therapies tailored to a certain gender. Furthermore, to explore the true association between protein intake and constipation, we adjusted for potential confounding factors that may be masked to ensure more reliable outcomes. Nevertheless, there are limitations. Being a cross-sectional study, it cannot establish causality or the direction of the relationship between constipation and protein intake. Also, the reliance on self-reported data from the NHANES database could introduce recall bias when investigating stool types and 24-h dietary recalls. At the same time, the NHANES database does not differentiate between types of protein in its assessment of protein intake. Therefore, we are unable to further analyze the relationship between different types of protein and constipation in men and women. Lastly, while NHANES offers extensive national health parameter estimates, incomplete data access restricted our ability to fully adjust for potential confounders. Despite these limitations, our findings lay the groundwork for future mechanistic and intervention studies, highlighting the need for prospective research to further validate and expand upon our conclusions.

## Conclusion

In summary, our study provides the first nationwide estimate of the relationship between dietary protein intake and constipation. We found that in men, there’s a negative dose–response relationship between dietary protein intake and the risk of chronic constipation. In contrast, after adjusting for confounding factors, women showed a correlation between higher dietary protein intake and an increased risk of chronic constipation. This difference between genders is statistically significant. Threshold effect analysis further clarified the gender-specific threshold of dietary protein intake to reduce the risk of chronic constipation. These findings offer new insights into managing chronic constipation across genders, but further research is needed to confirm and expand upon them.

## Data availability statement

Publicly available datasets were analyzed in this study. This data can be found at: https://www.cdc.gov/nchs/nhanes/.

## Ethics statement

The studies involving humans were approved by the National Center for Health Statistics Research Ethics Review Board. The studies were conducted in accordance with the local legislation and institutional requirements. The participants provided their written informed consent to participate in this study.

## Author contributions

YH: Writing – original draft. HS: Writing – original draft. XC: Writing – review & editing. GL: Writing – review & editing.
